# 14. Monkeypox viral detection from different body sites. Northern California, June–July 2022

**DOI:** 10.1093/ofid/ofac492.1865

**Published:** 2022-12-15

**Authors:** Krithika Srinivasan, Dora Y Yuk-Wai Ho, Caitlin A Contag, Philip Grant, Abraar Karan, Shanthi Kappagoda, Thomas Dieringer, Ashley Styczynski, Benjamin A Pinsky, Jorge Salinas

**Affiliations:** Stanford, Palo Alto, CA; Stanford University, Palo Alto, California; Stanford University, Palo Alto, California; Stanford University, Palo Alto, California; Stanford, Palo Alto, CA; Stanford University, Palo Alto, California; Stanford University, Palo Alto, California; Stanford University, Palo Alto, California; Stanford University, Palo Alto, California; Stanford University, Palo Alto, California

## Abstract

**Background:**

Unlike prior outbreaks of monkeypox, the 2022 outbreak is unique because lesions have been predominantly noted in the anogenital area, especially among persons identifying as gay, bisexual, or other men who have sex with men. The role of various body sites in monkeypox transmission is currently unclear. We assessed monkeypox PCR positivity from various body sites.

**Methods:**

We collected demographic, clinical, and laboratory data from patients with confirmed monkeypox at Stanford during June–July 2022. We obtained samples from skin lesions, oropharynx, saliva, conjunctiva, urine, semen, and rectum. Monkeypox DNA analysis was performed on site using a quantitative PCR modified from published CDC assays.

**Results:**

During June–July 2022, 10 patients were confirmed to have monkeypox. All were men; median age was 40 (range 24–46). Four patients identified as Hispanic, three as Caucasian, two as African American, and one as Asian. Additionally, three had HIV infection and the remaining seven were on HIV pre-exposure prophylaxis. At the time of diagnosis, eight patients (80%) described a prodrome of fever and malaise. Seven (70%) had genital lesions; the remaining three (30%) presented with only extra-genital lesions. Complications included two patients with proctitis (20%), one with phimosis (10%) and one with tonsillar involvement and impending airway compromise (10%). A total of seven (70%) received tecovirimat, and one was hospitalized. Median time from symptom onset to sample collection was seven days (range 3–14). All patients had at least one skin lesion sample positive for monkeypox virus DNA (Table 1). Six rectal samples (100%), Eight oropharyngeal samples (89%) and two (66%) urine samples were also positive.

**Figure:**
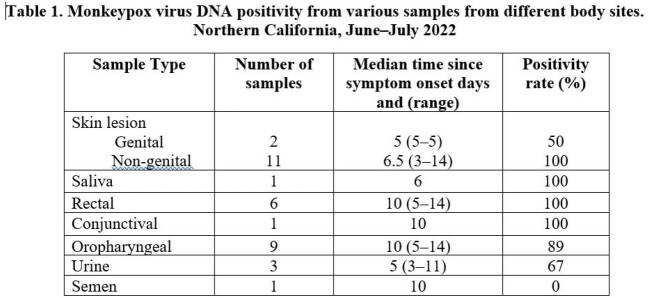

**Conclusion:**

We found monkeypox PCR positivity from different body sites. Public health messaging should consider targeting behaviors that involve body sites beyond skin, with particular consideration to mucosal surfaces.

**Disclosures:**

**All Authors**: No reported disclosures.

